# Endovascular therapy for cerebral vasospasm after aneurysmal subarachnoid hemorrhage: Single-center experience in a high-volume neurovascular unit

**DOI:** 10.1016/j.bas.2024.104133

**Published:** 2024-11-06

**Authors:** Carolin Albrecht, Raimunde Liang, Dominik Trost, Isabel Hostettler, Martin Renz, Bernhard Meyer, Claus Zimmer, Jan Kirschke, Christian Maegerlein, Jannis Bodden, Charlotte Lingg, Arthur Wagner, Tobias Boeckh-Behrens, Maria Wostrack, Julian Schwarting

**Affiliations:** a Department of Neurosurgery, Technical University of Munich, School of Medicine, Klinikum Rechts der Isar, Germany; b Department of Diagnostic and Interventional Neuroradiology, Technical University of Munich, Germany, School of Medicine, Klinikum Rechts der Isar, Germany; c Department of Neurosurgery, Cantonal Hospital St. Gallen, St. Gallen, Switzerland; d Department of Anesthesiology, Technical University of Munich, Germany, School of Medicine, Klinikum Rechts der Isar, Germany; e Institute for Stroke and Dementia Research (ISD), University Hospital, LMU Munich, Munich, Germany

**Keywords:** aSAH, Aneurysmal subarachnoid hemorrhage, CVS, Cerebral vasospasm, Subarachnoid hemorrhage, Vascular disorders, Delayed cerebral ischemia

## Abstract

**Introduction:**

Despite targeted standard therapy, aneurysmal subarachnoid hemorrhage (aSAH) frequently leads to cerebral vasospasms (CVS) of large cerebral arteries, reduced oxygen supply of brain tissue, known as delayed cerebral ischemia (DCI), subsequent development of manifest cerebral infarction and poor neurological outcome.

**Research question:**

The primary aim was to evaluate the efficacy of endovascular spasmolysis (eSL) as a rescue therapy for delayed ischemic neurological deficits (DIND) occurring despite maximum conservative treatment, with the potential benefit of preventing permanent ischemic deficits, and thus, improving overall neurological outcomes.

**Material and methods:**

In our retrospective, monocentric study, we included 310 patients developing CVS during hospitalization and evaluated their clinical and radiographic outcomes. Severe vasospasm was defined by a mean velocity of >200 cm/s in transcranial Doppler ultrasound and/or occurrence of new neurological deficits, and/or decrease of at least 2 points on the Glasgow Coma Scale (GCS), respectively.

**Results:**

92 patients (29.7%) underwent eSL due to persistent symptoms despite conservative therapy. Among endovascularly treated patients, 86% (n = 79) improved angiographically, 71% (n = 44) of 62 patients who underwent eSL due to symptomatic deterioration improved clinically. Clinical worsening due to progressive CVS occurred in 18% of cases (n = 11). Periprocedural complications were observed in 4% (n = 4).

**Discussion and conclusion:**

eSL emerges as a safe and effective therapy for individuals experiencing DIND triggered by large-artery vasospasm following aSAH. The implementation of a standardized, multi-step process for detection and management, coupled with criteria for endovascular interventions, proves to be an efficient preventative approach to enhance neurological outcomes after aSAH.

## Highlights

• Endovascular spasmolysis can act as rescue therapy for cerebral vasospasm, but evidence is limited.
• Endovascular spasmolysis improved neurological deficits in 71 % of individuals in this large retrospective cohort study.
• Standardized detection and management with well-defined criteria for endovascular interventions should be implemented.

## 1 Introduction

Aneurysmatic subarachnoid hemorrhage (aSAH) is a severe type of hemorrhagic stroke that results from a rupture of intracranial aneurysms (Hoh et al., 2023). Despite a global decline in the incidence of aSAH attributed to public health initiatives and lifestyle modifications, the associated mortality rates persist at elevated levels (Thompson et al., 2022).

Although technical advances enable an effective aneurysm occlusion, either through endovascular intervention or neurosurgical clipping, the neurological outcome depends, among others, on the occurrence of delayed cerebral ischemia (DCI) (Janardhan et al., 2006; Frontera et al., 2009). An important mechanism of DCI is the occurrence of cerebral vasospasm (CVS) in large intracranial arteries which can reduce cerebral blood flow (CBF) and potentially lead to infarction, poor clinical outcome, and death. CVS occurs in approximately 30% of patients, predominately between days 4 and 14 after aneurysm rupture, and is known to be the most common cause of acute focal cerebral ischemia after aSAH (Janardhan et al., 2006; Molyneux et al., 2005; Spetzler et al., 2015). Large amounts of cisternal blood, graded by the modified Fisher or the Barrow Neurological Institute (BNI) scales, is an established predictor of CVS and DCI after aSAH (Neidert et al., 2018; Frontera et al., 2006). Other commonly known risk factors are young age and female sex (Wilson et al., 2012; Charpentier et al., 1999).

The diagnosis and therapy of CVS varies substantially between centers. The amount of prospective multicenter trials is limited. At the moment, the only proven effective prophylactic measures against CVS are enteral application of the calcium antagonist Nimodipine and maintaining euvolemia (Allen et al., 1983; Macdonald and Schweizer, 2017). This is in line with the current guidelines for the management of patients with aSAH (Hoh et al., 2023). Therapeutic approaches currently involve induced hypertension, as triple H therapy has fallen out of favor due to its associated hemodilution-related adverse effects (Muench et al., 2007; Lennihan et al., 2000; Guresir et al., 2022). However, the prematurely halted HIMALAIA trial did not provide sufficient evidence to support induced hypertension and highlighted the potential risk of significant adverse events (Gathier et al., 2018).

Endovascular treatment of CVS, either by local intraarterial application of calcium antagonists or by mechanical dilatation of focal stenoses, has become an additional therapeutic option in selected cases (Eskridge et al., 1998; Firlik et al., 1997). However, the only available prospective, randomized, multicenter trial reported less favorable outcomes after 6 months follow-up in patients who were randomized to endovascular CVS treatment (Vatter et al., 2022). Despite these less favorable results, eSL is still widely practiced in neurovascular centers, including ours. The aim of this study was to evaluate whether eSL is indeed effective in preventing DCI. Based on past observations, certain patient subgroups may benefit from this treatment, and our goal was to identify which patients may respond better to eSL.

Bearing in mind the controversial evidence for the effectiveness of the treatment, we analyzed the angiographic and clinical results of a large cohort of patients treated by endovascular intervention (eSL) in our high-volume center.

## 2 Methods

### 2.1 Study design

This cohort study was designed in accordance with the STROBE guidelines (von Elm et al., 2007). We retrospectively examined the medical data of all patients who were treated at our center for aSAH between January 2006 and March 2020.

Patients were included to the analysis based on the following criteria.

1. Admission with acute aSAH between January 2006 and March 2020.
2. Ruptured aneurysm, confirmed by CT-angiography or digital subtraction angiography (DSA) that was occluded at our center.

**Fig. 1:**
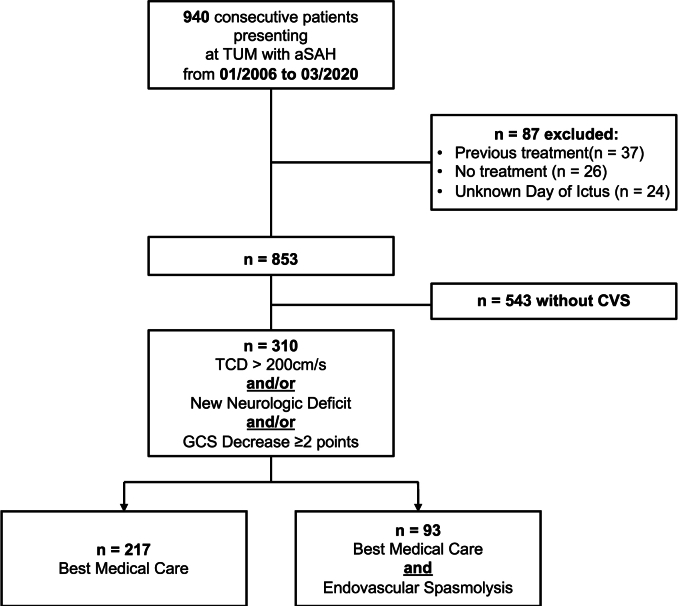
– Cohort diagram.

Patients with non-aneurysmatic SAH, patients who were primarily treated elsewhere, or patients treated later than 14 days after aneurysm rupture were excluded from the analysis (the cohorts are displayed in Fig. 1).

### 2.2 Definition of CVS and conservative therapy

Severe CVS was defined as either an increase of the mean blood flow velocity (V_mean_) in the middle cerebry artey (MCA), anterior cerebral artery (ACA), or internal carotid artery (ICA) to >200 cm/s in transcranial Doppler ultrasound (TCD) and/or occurrence of a new focal neurologic deficit and/or decrease of at least 2 points on the Glasgow Coma Scale (GCS) after exclusion of other potential reasons such as but not limited to postoperative hematoma, hydrocephalus, metabolic imbalances, or seizures (Vergouwen et al., 2010).

**Fig. 2:**
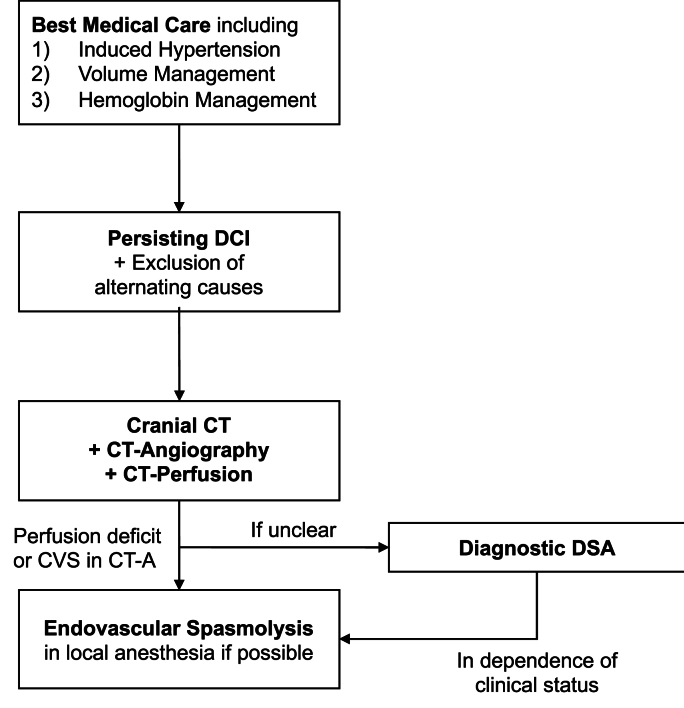
– Internal clinical standard scheme.

Standard prophylactic treatment included enteral administration of 60 mg of Nimodipine every 4 h for all patients with aSAH for 14 days starting from the day of the aneurysm treatment. TCD examination was done daily during the first 14 days after aSAH or longer, until the definitive decrease of the flow velocity, in the rare cases of a prolonged CVS phase. Conservative measures in case of diagnosed CVS were applied immediately and included induced hypertension, mostly using noradrenalin, euvolemia under consistent hemodynamic monitoring and optimization of the hemoglobin values. Decisions for eSL were met in accordance with the internal clinical standard scheme, summarized in Fig. 2.

### 2.3 Clinical data

The severity of the initial bleeding event was categorized according to the Hunt & Hess (Hunt and Hess, 1968) and WFNS (Rosen and Macdonald, 2005) grading systems, as well as the Glasgow coma scale (Teasdale and Jennett, 1974). The past medical history of patients was acquired through medical files and questionnaires of patients and relatives. The neurological status was recorded hourly during the first 14 days after aSAH.

Outcome after eSL was dichotomized into “partly improved”, “fully improved”, “unchanged”, and “worsened”, respectively, and was accessed immediately after the intervention, during the subsequent course, and at discharge. For neurological outcome at discharge we used a dichotomized version of the modified Rankin Scale (mRS) (Rankin, 1957).

### 2.4 Radiological data

Radiographic data was acquired from radiological files and reports. The extent of cisternal blood clot was characterized by the modified Fisher (mFisher) score (Frontera et al., 2006). Aneurysms were described by size and location. Locations were categorized into MCA, ACA, anterior communicating artery (ACOM), posterior communicating artery (PCOM), and ICA. Location in the posterior circulation (basilar artery, posterior inferior cerebellar artery, anterior inferior cerebellar artery, superior cerebellar artery and vertebral artery) was pooled as such due to the small numbers. Angiographic improvement was defined as an immediate enlargement of the vessel lumen, with a dilation of 15% or more considered significant.

### 2.5 Endovascular CVS treatment

ESL was performed under local or general anaesthesia with continuous surveillance of the blood pressure by invasive measurement. A 5F transfemoral or transradial arterial access was established, and 5F diagnostic catheters were navigated to the ICA and VA. Treatment was then started in the most affected vascular territory while continuously monitoring symptoms, in the case of awake patients, and the effect on the angiogram.

Intraarterial, medical spasmolysis was performed. Depending on the systemic effect on the mean arterial blood pressure, up to 2.5 mg of Nimodipine were injected per target vessel over 30 min 3000–5000 IU of heparin were given simultaneously to prevent thromboembolic complications (Guenego et al., 2023). In cases of proximal focal stenoses, mechanical treatment was performed: Percutaneous Transluminal Angioplasty (PTA) via balloon (preferably non-compliant) or other devices, i.e., Comaneci (Rapid Medical) (Eskridge et al., 1998; Salem et al., 2023).

### 2.6 Decision for eSL

The indication for eSL was made interdisciplinary, primarily based on the occurrence of Delayed Ischemic Neurologic Deficits (DIND) in combination with CT-perfusion and/or CT-angiography, aligned with the core principles of the Vergouwen criteria, ensuring that our diagnostic and treatment process reflects established standards in identifying and managing DIND and DCI (Vergouwen et al., 2010). DIND was characterized by either focal neurological deficits (such as hemiparesis, aphasia, hemianopia, or neglect) or global impairment (such as a two-point reduction on the Glasgow Coma Scale) lasting at least 1 h and/or cerebral infarction, whereas other possible causes of clinical deterioration, e.g. hydrocephalus, seizures, rebleeding, or hyponatremia, were excluded (Vergouwen et al., 2010). In case of a clinical recurrence of vasospasm after endovascular intervention, the treatment was repeated (Guenego et al., 2023). The decision on repeated treatment in case of the deficit's recurrence was based on the evidence of the immediate postinterventional clinical success, the absence of irreversible ischemic changes on follow up CT scans, and interdisciplinary discussion between the treating neurosurgical and neuroradiological departments. Patients who showed clear clinical and radiologic improvement after eSL, but later experienced secondary neurological deterioration attributed to CVS, were scheduled for repeat intervention following interdisciplinary consensus.

## 4 Results

### 4.1 Patient characteristics

853 patients (568 females, mean age 57 ± 14 years) with aSAH were included in the study (Fig. 1). Clinical severity, according to the Hunt and Hess classification, was: Grade II (34%) > Grade III (22%) > Grade IV (18%) > Grade I (14%) > Grade V (13%). Most patients had a mFisher grade 3 on the initial CT scan (88%, n = 268, Table 1).

**Table 1:** – Baseline characteristics and univariate comparisons of patients with and without endovascular intervention (eSL).

Parameter		No SL (n = 218)	eSL (n = 92)	Total (n = 310)	p
**Age**		53 ± 13	55 ± 12	54 ± 13	0.516
**Female sex**		161 (70%)	62 (77 %)	223 (73%)	0.182
**Hunt & Hess**	**1**	20 (9 %)	10 (11 %)	30 (10 %)	0.695
	**2**	77 (35 %)	26 (28 %)	103 (34 %)	
	**3**	43 (20 %)	23 (25 %)	66 (21 %)	
	**4**	46 (21 %)	21 (23 %)	67 (22 %)	
	**5**	32 (15 %)	12 (13 %)	44 (14 %)	
**mFisher**	**1**	5 (2 %)	3 (4 %)	8 (3 %)	0.916
	**2**	14 (7 %)	6 (7 %)	20 (7 %)	
	**3**	190 (89 %)	78 (87 %)	268 (88 %)	
	**4**	5 (2 %)	3 (4 %)	8 (3 %)	
**Aneurysm site**	*ICA*	21 (10 %)	12 (13 %)	33 (11 %)	0.227
	*ACA*	8 (4 %)	4 (4 %)	12 (4 %)	
	*MCA*	72 (33 %)	21 (23 %)	93 (30 %)	
	*ACOM*	55 (25 %)	34 (37 %)	89 (29 %)	
	*PCOM*	34 (16 %)	12 (13 %)	46 (15 %)	
	*Post. Circ.*	28 (13 %)	9 (10 %)	37 (12 %)	

**Clipping**		101 (43%)	31 (33 %)	132 (46 %)	**0.040**

**Mean V**_**mean**_		257 ± 50	267 ± 59	260 ± 53	0.117
**Minimal V**_**mean**_		100	110	100	
**Maximal V**_**mean**_		400	400	400	
**Spasm onset in days after SAH**		8 ± 8	9 ± 5	8 ± 8	
**New infarcts**		50 (23%)	42 (46%)	92 (30%)	**< 0.001**

**Hospital duration**		27 ± 12	33 ± 19	29 ± 15	**0.003**
**Death < 30d**		23 (11%)	12 (14%)	35 (12%)	0.369

**6 months mRS**	n^*‡*^	172 (79%)	70 (76%)	242 (78%)	**0.028**
	0–2	106 (62%)	30 (42%)	136 (56 %)	
	3–5	41 (24%)	31 (44 %)	72 (30 %)	
	6	25 (15%)	9 (13 %)	34 (14 %)	

*Note. —mFisher = modified Fisher Scale. mRS = modified Rankin Scale. N*^*‡*^ = *Number of patients followed up after* 6 months*.V*=*Velocity*.

Of the initially screened 853 patients, 310 (i.e. 36%) had CVS according to our definition. 92 patients received endovascular spasmolysis (eSL).

Patients undergoing eSL did not significantly differ from patients with CVS who did not undergo eSL in terms of age, sex, Hunt & Hess score, mFisher score, aneurysm site, aneurysm occlusion method, and V_mean_ in TCD **(**Table 1**).** Patients who underwent eSL in our cohort appeared to be less frequently treated by surgical clipping as compared to coiling (33% vs. 43%, p = 0.04, Table 1**).**

### 4.2 Overall outcome

While mortality was not significantly different between groups, patients who received eSL had a longer hospital stay (p = 0.003) and significantly worse neurological outcome measured by the dichotomized mRS score 6 months after aSAH (p = 0.028).

Also, patients undergoing eSL were about twice as likely to suffer from cerebral infarcts during the course of the disease (46% vs. 23%, p < 0.001, Table 1). Most patients (48%) exhibited a new onset hemiparesis. In 33% of cases, the indication was based on an increased V_mean_ only, e.g., in patients who could not be examined due to coma or deep sedation (Table 2).

**Table 2:** – Indication for endovascular Spasmolysis.

		n = 92	%
**Clinical**	**Total**	62	67
	**Hemiparesis**	30	48
	**Aphasia**	11	18
	**Other neurological deficit**	7	11
	**Decrease in GCS**	15	24

**Only TCD >200 cm/s**	**Total**	30	33

*Note. — GCS = Glasgow coma scale. TCD = Transcranial Doppler Sonography of intracranial arteries*.

### 4.3 Endovascular interventions, outcome, and complications

Overall, 241 interventions were performed in 92 patients. CVS predominantly occurred in the anterior circulation. Most patients were treated with local intraarterial Nimodipine alone (n = 63), whereas 19 were treated by combining percutaneous transluminal angioplasty (PTA) and Nimodipine. 44 (48%) patients underwent multiple sessions of eSL due to persistent or recurrent CVS. Angiographically, vasospasm was reduced in 86% of cases. The incidence of intervention-associated complications remained low, with 1 extracranial and 2 intracranial vessel dissections and 1 groin pseudoaneurysm in 241 interventions, resulting in an overall risk of 1.7% (Table 3).

**Table 3:** – Results of endovascular intervention.

		n = 92	%
**Type of Intervention**	**Local Nimodipine**	63	68
	**Local Nimodipine + PTA**	19	21
	**PTA**	10	11

**Affected Vessel**	**Unilateral anterior circulation**	25	27
	**Bilateral anterior circulation**	25	27
	**Posterior circulation**	4	4
	**Generalized**	38	41

**Interventions/patient**	**Median**	1	
	**Minimum**	1	
	**Maximum**	14	

**Angiographic Result**	**Improvement**	79	86
	**No improvement**	13	14

**Complications**	**Intracranial dissection**	2	0.8
	**Extracranial dissection**	1	0.4
	**Groin complication**	1	0.4

Note. — GCS = Glasgow coma scale. PTA = percutaneous transluminal angioplasty.

### 4.4 Symptomatic conscious patients

In 44 (71%) of the 62 patients who underwent eSL due to a clinically apparent deterioration, neurologic symptoms improved after the last endovascular intervention and remained stable during the rest of the hospital stay in case of multiple eSL sessions. Symptoms remained unchanged in 11 patients without any subsequent worsening. Neurological symptoms worsened in 7 patients despite eSL; 6/7 of these patients developed infarcts during the hospital stay (Table 4).

**Table 4:** – Clinical results of endovascular intervention.

Symptomatic conscious patients		n = 62	%
**Hunt & Hess Grading**	**I**	9	15
	**II**	24	39
	**III**	15	24
	**IV**	10	16
	**V**	4	6
**Overall clinical status**	**Improvement**	44	71
	**Stable**	11	18
	**Worse**	7	11
**Secondary infarct after eSL**		21	33
**Death before discharge**		4	6


**Unconscious patients**		**n = 30**	**%**

**Hunt & Hess Grading**	**I**	1	3
	**II**	1	3
	**III**	8	27
	**IV**	12	40
	**V**	8	27
**Secondary infarct after eSL**		25	83
**Death before discharge**		8	27

Note. — eSL = Endovascular intervention.

TCD = Transcranial doppler.

### 4.5 Unconscious patients

In 30 patients, clinical examination was not possible due to an unconscious state or required sedation. These patients received eSL based on elevated TCD flow. Treatment success was measured using surrogate parameters: V_mean_ in TCD and the subsequent occurrence of new infarcts in the treated vessel territories. Notably, we observed a rise in V_mean_ in 37% of cases (n = 11) after eSL, and a substantial 83% (n = 25) of patients experienced the development of new infarcts following eSL. (Table 4).

### 4.6 Multivariable prediction of clinical improvement after eSL among awake patients at the onset of DIND

An analysis of factors predicting favorable outcomes after eSL among those who were awake did not show significant differences in age, sex, mFisher grades, Hunt & Hess grades, aneurysm occlusion method, or aneurysm site. (Suppl Table 1).

## 5 Discussion

In this retrospective analysis within a large cohort of aSAH patients with CVS, a distinct favorable effect of eSL was evident among patients with unimpaired baseline consciousness, where 71 % of individuals with newly onset deficits experienced immediate and sustained resolution. However, for unconscious patients with a less clearly monitorable clinical state, no discernible benefit was observed.

Endovascular therapy for CVS, involving Nimodipine or mechanical dilatation, yields effective angiographic results (Jun et al., 2010). Intraarterial Nimodipine is particularly effective in proximal and distal vessel segments but carries a risk of recurrence. Mechanical dilatation is limited to proximal lesions and is most effective in refractory cases and isolated focal stenoses. Despite it being rare, it is crucial to recognize that some patients may experience clinical deterioration despite technically effective endovascular intervention.

While several retrospective studies suggest benefits of endovascular spasmolysis in individual clinical settings, the only available randomized multicenter study failed to demonstrate clinical benefits of interventional treatment compared to best medical care alone (Jun et al., 2010; Vatter et al., 2022). Moreover, they observed worse outcomes, when comparing eighteen patients treated conservatively with sixteen patients treated endovascularly. Outcome measures included the extent of new ischemic lesions, as apparent in MRI DWI sequences, and mRS at 6 months after discharge. In addition, they observed an increased rate of periinterventional complications compared to the standard medical treatment (Vatter et al., 2022). In line with the randomized study, we also observed worse mRS scores in patients who underwent endovascular treatment for severe vasospasms as compared to those with conservative treatment only. However, this is obviously related to the initially poorer baseline condition of the patients undergoing spasmolysis due to severe symptomatic CVS and therefore making them more susceptible to secondary brain injury - a clinical problem that intervention alone may not entirely eliminate. Furthermore, in our patient cohort, a significant rate of newly occurred cerebral infarcts was observed similarly to the randomized trial. Most CT findings did not correlate with clinical deficits. Clinical improvement occurred despite new infarcts, probably due to their small size and non-eloquent location. Notably, immediate and sustained clinical improvement was observed in 71% of patients with good baseline conditions. Only 11% experienced further neurological impairment, primarily due to otherwise uncontrollable ischemic development. Remarkably, patients who underwent eSL were already receiving the maximum conservative regimen as per the clinic's internal standard operating protocol.

A recent study published by Vossen et al. analyzed the outcome of 96 patients receiving intra-arterial nimodipine upon development of DCI unresponsive to induced hypertension. The data indicated a low rate of complication associated with IAN and a good outcome for approximately half of the patients receiving IAN. Consistent with our study, they also presented ambiguous results in severely disabled aSAH patients (Vossen et al., 2024). The benefit for comatose patients in our study cohort remained uncertain and was not clearly apparent by our results. A possible explanation is that the occurrence of DCI may not be adequately captured by TCD alone and may be caused by factors other than large-artery CVS. Furthermore, the delayed detection of CVS in comatose patients may lead to delays in rescue therapy, and immediate therapeutic effects are challenging to capture. Early identification of at-risk patients, coupled with shorter TCD intervals or pTiO2 and microdialysis probes, could potentially improve an earlier detection of changes (Gouvea Bogossian et al., 2023). However, additional early brain injury mechanisms, including microcirculatory dysfunction or inflammatory responses, along with secondary complications, such as cardiopulmonary issues, hydrocephalus, or electrolyte disorders – commonly observed in severely affected SAH patients – appear to be the main contributor to the occurrence of secondary infarcts, DCI, and overall unfavorable patients’ outcomes (Lauzier et al., 2023; Schwarting et al., 2023a, 2023b). These could potentially be captured using invasive neuromonitoring approach, such as intraparenchymal probes or microcatheter, which were not established at our center during the study period for routine use (Falcone and Chen, 2022). A larger study focusing on SAH patients with higher Hunt and Hess grades is needed to confirm our hypothesis that cerebral vasospasm is difficult to treat in patients with poor clinical status, for whom endovascular spasmolysis may not be the appropriate therapy.

### 5.1 Limitations

Our study does have some limitations. Firstly, the study's monocentric, retrospective, and single-center design introduces inherent limitations. Additionally, utilizing V_mean_ > 200 cm/s as a surrogate parameter for DCI could be unreliable, particularly in unconscious patients. Moreover, the absence of longer follow-up data precluded the assessment of long-term neurological outcomes. Lastly, the challenging task of precisely measuring the timespan between the onset of CVS and eSL may potentially have an influence on the observed treatment results.

In our cohort, patients undergoing eSL appeared to be treated less frequently with surgical clipping compared to coiling (33% vs. 43%, p = 0.04, Table 1). We believe this difference may be due to random variation, as our study was underpowered to detect a significant difference between the two treatment modalities. This observed imbalance likely reflects limitations in statistical power rather than a meaningful clinical distinction between clipping and coiling.

This study is of a retrospective nature, which limits the strength of the conclusions that can be drawn. A more robust analysis would require a prospective study and specific subgroups to provide more definitive insights, as outlined in the IDEAL (Idea, Development, Exploration, Assessment, Long-term study) framework (Seidel et al., 2015). According to this approach, future studies should focus on defined subgroups to assess intervention outcomes and safety, which cannot be fully achieved in retrospective analyses such as ours. Currently, no such prospective studies exist for relevant subgroups of the aSAH cohort, as previous research has generally considered the entire aSAH population.

Additionally, there is no randomized control group only treated conservatively. Hence, the potential clinical outcome following continued conservative treatment remains uncertain. Nevertheless, it is noteworthy that all subjects in our study had already reached the threshold of maximal conservative therapy, indicating that further optimization of therapy was not feasible.

Based on all the above-mentioned observations, we recommend including eSL as an optional rescue therapy for patients with good baseline clinical status who exhibit DIND despite maximum conservative treatment, as it appears to be both efficient and safe for this subgroup. A multi-step decision protocol with clear criteria for eSL is essential to ensure its appropriate application, optimize patient selection, and maximize therapeutic outcomes while minimizing potential risks.

## 6 Conclusion

Our study shows in accordance with previous research that with standard conservative care, including oral nimodipine, induced hypertension, and careful management of volume and hemoglobin, therapy-resistant CVS occurs in about one-third of cases. While not clearly supportive for patients with poor clinical status, Endovascular spasmolysis appears to provide immediate and significant clinical benefit as a rescue therapy for acute DIND occurring despite maximum conservative treatment in selected cases, particularly those with good baseline clinical status. Large prospective studies focusing exclusively on the relevant target subgroups are needed to validate these findings.

## 3 Ethical approval

The study was approved by the local Research Ethics committee of the Technical University of Munich (186/20S). As this was a retrospective analysis and none of the included data harbors the possibility of being tracked back to a specific patient, the Research Ethics committee of the Technical University of Munich deemed our study exempt for retrospective informed consent for the included patients. All methods were performed in accordance with the relevant guidelines and regulations.

### 3.1 Statistical analyses

Data sets were tested for normal distribution with the D'Agostino & Pearson test and presented as mean ± standard deviation (SD) in case of normal distribution. In the context of data not adhering to a normal distribution, descriptive statistics such as the median and interquartile range (IQR), or alternatively, the minimum and maximum values are provided. Statistically significant differences between groups were tested with student's t-tests for parametric data and with the Mann-Whitney test for non-parametric data using Prism 8 (Graphpad Software LLC, USA) with a two-sided significance level (p) of 0.05. The logistic regression model was calculated with STATA v13.1 (StataCorp LLC).

## Availability of data and materials

The datasets generated during and/or analyzed during the current study are available from the corresponding author on reasonable request.

## Author contributions

All authors made substantial contributions to the conception, design, data acquisition, data analysis, and data interpretation. DT, IH, CA, MW acquired data. JB performed statistics. JS, CA, MW and TB-B drafted the manuscript and all other authors revised it critically and made substantial contributions. All authors approved the final version to be published.

## Declaration of generative AI and AI-assisted technologies in the writing process

During the preparation of this work the authors used *ChatGPT 4o* to improve English readability. After using this tool, the authors reviewed and edited the content as needed and take full responsibility for the content of the publication.

## Declaration of competing interest

The authors declare that they have no known competing financial interests or personal relationships that could have appeared to influence the work reported in this paper.
